# Treatment of Scabies

**Published:** 1851-11

**Authors:** 


*Treatment of Scabies.*—M. Bazin, physician to the Hôpital St. Louis, of Paris, published in July, 1850, several cases of itch cured in a few days by frictions with the sulphur ointment all over the body. The same physician confirms, in *L'Union Medicale* of November 9, 1850, the efficacy of the treatment, by stating,—"The patients who formerly left the hospital after twelve or fifteen days only, and very often came back with a relapse of the disease, are now cured in two or three days." But this is not all, for M. Bazin, finding out that some patients have an insurmountable dislike to sulphur, was induced to try several ointments prepared with essential oils—as those of lavender, thyme, rosemary, and likewise turpentine and mercurial ointment. None of these were effectual. He then had the idea of trying unmedicated axunge of oil, and found it answer remarkably well, six frictions during three days being sufficient.

It will be recollected that Mr. Taylor, surgeon to the Clerkenwell Infirmary, published a book some months since (see The Lancet, October 12, 1850, p. 418,) wherein he advocates frictions of a like character for the cure of typhus fever, scarlatina, &c. It would be highly useful if frictions, with simple unctuous substances, were more extensively tried in the diseases spoken of by Mr. Taylor and M. Bazin. The ease with which the remedy is obtained, and the little risk which is incurred by its use, would render it inestimable if found efficacious, especially as regards the treatment of the poor. M. Bazin has likewise tried a chamomile ointment, which cures the itch in three frictions. It is composed as follows: Powder of fresh chamomile, white oil, axunge, of each sixteen ounces. M. Bazin proposes, therefore, in ordinary cases, that the sulphur ointment be used, (sublimated sulphur, very fine powder, of each two drachms; one yolk of egg; olive oil one ounce and two drachms and a half,) in exceptional cases, either three frictions with the chamomile ointment, or six with the simple oil and axunge.—*Ibid.*

